# Items

**Published:** 1898-05

**Authors:** 


### ITEMS.

The Journal Advertising Agency, of which J. MacDonald, Jr., is manager, has removed to the Woodbridge Building, 100 William street, New York.

---

Victor Koechl & Co., importers of medicinal preparations, such as antipyrin, lanolin, Behring's antitoxin, argonin, orthoform, etc., etc., announce their removal from 79 Murray street to the new and modern six-story building, 122 Hudson street, corner of N. Moore street. The necessity of obtaining larger and more commodious quarters and better shipping facilities is the reason for making this change.

---

The name of Dr. Allen A. Jones was inadvertently omitted from the round-table group (p. 780) at the alumni association banquet of the medical association of Buffalo.

---

Medical Excursion in June.—(Denver to Salt Lake City.)—The medical excursion in June will leave Denver for Salt Lake City—the Zion of the new world—on the last day of the meeting of the American Medical Association and the two successive days via the Rio Grande Western Railway in connection with the D. & R. G. and Colo. Mid. lines. The rate will be but $18.00 for the round trip, offering a trip of 1,500 miles through the Rocky and Wasatch Mountains. No European trip of equal length can compare with it in grandeur or wealth of novel interest. Salt Lake City and vicinity is one grand sanitarium. The Great Salt Lake or Dead Sea of America with its magnificent bathing resort, the hot and warm springs, drives, parks, canyons and reserves are all located in or about the city. Send 2 cents to F. A. Wadleigh, Salt Lake City, for copy of pamphlet.

